# Assessing the Asphalt Binder Film Thickness in Recycled Asphalt Mixtures Using Micro-Level Techniques

**DOI:** 10.3390/ma14247891

**Published:** 2021-12-20

**Authors:** Fazli Karim, Jawad Hussain

**Affiliations:** Taxila Institute of Transportation Engineering, University of Engineering and Technology, Taxila 47050, Pakistan; jawad.hussain@uettaxila.edu.pk

**Keywords:** asphalt binder film, asphalt mastic, recycled asphalt mixtures, image analysis, analytical film thickness models

## Abstract

Adequate asphalt binder film thickness (ABFT) delivers skeletal integrity in recycled asphalt mixtures, resulting in long-lasting roadways when exposed to traffic and environment. The inaccurate measurement of ABFT and the consequences of not having adequate film thickness model has substantially introduced discrepancies in predicting actual performance of recycled asphalt mixtures. Expansion of the ultra-modern expertise and SuperPave requirements necessitate the revision of authentic ABFT at micro-level. The current study identifies the weaknesses of the current methods of estimating ABFT and provides results that are reliable and useful, using modern measurement methods. Using scanning electron microscope (SEM) and energy dispersive x-ray spectroscopy (EDS), this study measures the ABFT around the tiniest particle of 0.2 μm magnitude, entrenched in asphalt mastic in recycled asphalt mixtures. The ABFT, obtained through image analysis, is compared with those obtained through available analytical models. The study utilizes different asphalt mixtures, containing varying proportions of recycled asphalt mixture and rejuvenators. The aggregate, virgin, and recycled binders were characterized in terms of physical and rheological properties, respectively. Marshall mix design was carried out for the conventional and recycled mixture, containing 40%, 50%, and 60% recycled materials, rejuvenated with 3%, 6%, 9%, and 12% waste engine oil (WEO) at a mixing temperature of 160 °C, based on viscosity of the virgin and rejuvenated binder. ABFT was assessed through analytical models and image analysis for the aforesaid recycled asphalt mixtures, prepared at optimum binder and rejuvenator content as per protocol outlined in ASTM D1559. The analytical estimation of ABFT, in the aforesaid recycled asphalt mixtures, revealed that the ABFT fluctuates from 6.4 μm to 13.7 microns, with a significant association to recycled asphalt mixture and rejuvenator content. However, the image analysis revealed that the ABFT, in the aforesaid recycled asphalt mixtures, fluctuates from 0.4 μm to 2 microns, without any association to recycled asphalt mixture or rejuvenator content. The image analysis indicated that the recycled asphalt mixtures typically comprise of mortar, happening in uneven shape, and are used to grip large aggregates. The asphalt mastic, a blend of bitumen and mineral filler, was found to be an interlocking agent, used to grasp only fine particles in asphalt mortar. The asphalt binder film was discovered to be a deviating stand-alone entity that only exists around the mineral fillers in the asphalt mastic as a non-absorbed binder, occupying an imprecise space of 0.4 μm to 2 microns, among the filler particles. The current findings will be useful to design asphalt pavements through the aforesaid precise limit of SEM-based ABFT rather than traditionally measured ABFT to predict the actual performance of recycled asphalt mixtures.

## 1. Introduction

Many factors influence the durability of hot mix asphalt (HMA), but volumetric features, such as voids in mineral aggregates (VMAs) and air voids (AV), are important. Since the late 1950s, the lowest VMAs have been employed as a design criterion in asphalt mixtures, but difficulties in attaining VMAs in Superpave^®^ mix design have prompted various new investigations. As a result, rather than using the minimal VMAs in Superpave volumetric mix design, some researchers advocate using the average asphalt binder film thickness (ABFT) as a design criterion in asphalt mixtures [1,2]. The thickness of the effective asphalt binder utilized to efficiently form a coat on the aggregate surface in HMA is referred to as ABFT. The ABFT around the aggregate in HMA is also known as an interface bonding agent or adhesion promoter. The prime objective of ABFT is to ensure structural integrity among the aggregates in HMA so that they may perform as a single system to bear wheel loads. The inadequate ABFT, on the other hand, will promote aggregate clustering or agglomeration in the HMA. Each cluster will function as an independent system, affecting structural integrity in the aggregate, perhaps speeding up the rate of HMA degradation when exposed to traffic and the environment. The skeletal cohesion and adhesion of asphalt paving mixtures are based on the ABFT around the granular particles, which affects HMA durability [3,4,5].

A broad notion suggested that an average ABFT thickness of 8 to 15 μm would offer satisfactory pavement performance over time [6]. The minimum ABFT of 6 to 8 μm is generally recommended for recycled asphalt mixtures, and virgin HMA is considered adequately serviceable. However, no relevant literature data are available to check the existence of the aforesaid values of ABFT. Apart from introducing complexities in achieving VMA in asphalt mix design, the thickness of ABFT around the aggregate in compacted paving mixtures is very important to be considered. It is remarkably noted that coarser HMA, due to lower surface area, has a substantial thick binder film around the aggregate but usually flops to meet the criteria of minimum VMAs. Likewise, fine HMA, due to higher surface area, has lower binder film and fulfils the minimum VMA criteria. As a result, for the mix design of recycled asphalt mixtures and virgin HMA using the Superpave method, the minimum VMA requirement is proposed to be based on minimum ABFT rather than minimum asphalt content [2]. The VMA criterion for coarse aggregate gradation is similarly difficult to achieve in the field [7]. Furthermore, when designing an open grade asphalt mixture, the air voids percentage of the compacted mixture is typically greater than 18%. As a result, the open grade asphalt mixture’s durability cannot be guaranteed by the minimum VMA index [8].

Using a modified micrometer, a test method was developed to investigate a thin coating of asphalt binder between a cylindrical rock core and a metallic stub with a regulated geometry. In asphalt mixture design, the approach of “density grading mixture” was employed to compute average asphalt binder film thickness, which ranged from 8 to 10 µm [9]. On the other hand, the effect of asphalt binder film thickness on asphalt pavement performance is usually neglected, especially when performance falls short of expectations. As a result, a full understanding of the effect of ABFT on the mechanical properties of compacted asphalt mixtures is required [10]. To investigate the concept of asphalt binder film thickness and its relationship to mixing temperature and binder content in conventional asphalt mixtures using SEM and EDS, F. Karim et al. [10] produced Marshall Specimens at mixing temperatures of 140, 150, and 160 °C. The study revealed that the film thickness is a function of temperature, binder concentration, and model type, according to the estimated values of asphalt binder film thicknesses using analytical models. A substantial variance in asphalt binder film thickness was reported for mix design at constant binder concentration and mixing temperature. Analytical calculations demonstrated that the asphalt binder film thickness varied from 9 to 13 µm under all temperature and binder content conditions, with a fair relationship between binder content and mixing temperature. However, imaging analysis revealed that the thickness of the asphalt binder film varied from 0.5 to 2.4 µm with no correlation to binder concentration or mixing temperature.

Kandhal and Chakraborty discovered a substantial relationship between asphalt binder film thickness, the tensile strength, and resilient modulus of HMA using conventional film thickness calculations [11]. As a result, they recommended using an average film thickness of 9 to 10 µm for specimens compacted with 8% air voids.

Attempts are made to replace the minimum VMA requirement in asphalt mix design with appropriate film thickness, resulting in diverse ABFT values for optimal HMA performance, as shown in Table 1.

Elseifi et al. [18] used scanning electron microscopy to study the notion of asphalt binder film thickness in hot mix asphalt. The experimental program’s findings were used to better understand the idea of asphalt binder film thickness and its validity. According to the findings, the asphalt binder films in the mastic were found at a thickness of 2 µm. In order to study the concept of binder film thickness, Marshall Specimens with 50% virgin mixture and 50% recycled asphalt mixture at three mixing temperatures, 140 °C, 160 °C, and 180 °C, were prepared. The specimens were named A140, A160, and A180. Binder film thickness was measured utilizing electron microscopy and image analysis techniques on the specimens. To identify the recycled binder distribution, titanium dioxide was introduced as a tracer to virgin bitumen. The sample A140, which was mixed at 140 °C, the most common film thickness found was 3 µm, with the binder film ranging from 1.5 to 6 µm, indicating a tendency for thicker recycled binder film. For all of the samples examined, Sample A160, mixed at 160 °C, displayed similar results, ranging from 2.5 to 4.5 µm. The recycled binder film thickness in Sample A180 is less than in the other two samples, resulting in a distinct behavior. The film thickness for this mixture was between 1.5 and 3 µm. It was observed that the most frequent value of recycled binder film thickness in sample A180 is 2 µm, whereas greater recycled binder film values, such as 4–6 µm, were seen in the other two samples but not in sample A180 [19].

Traditional ABFT estimation in virgin and recycled asphalt mixtures depends on the number of analytical methods, as shown in Table 2, which were employed in the current study to estimate average ABFT in recycled asphalt mixtures.

Where:

FT_b_, T_F_, DA, T_f_, F, and F_be_ stand for ABFT, which is measured in μm (10^−6^ m) or (μm).

These models are based on assumptions, such as sphere-shaped aggregates, undeviating ABFT on them, and no clarification of the magnitude of compaction or porosity of the asphalt mixture, among other factors, which could be the source of inaccuracies in traditional ABFT estimation. Despite the fact that all particles in the mixture are unlikely to be covered with the same undeviating film thickness, this idea has been presumed to be true, despite the fact that there are no experimental results to support the use of this attribute. Furthermore, it is debatable if the ABFT is evenly distributed in the asphalt mixture. As a result, micro-level approaches must be used to investigate the concept of asphalt binder film thickness in asphalt mixtures. When looking at the ABFT values in Table 1, it is clear that there is a lot of variability in ABFT for the optimal HMA performance. Therefore, it is very problematic to track a precise limit of ABFT, to be followed for the preeminent performance of recycled asphalt mixtures. To the author’s knowledge, the majority of ABFT research has been theoretical, with only a few attempts to measure this property experimentally. As a result, the current research aims to examine the magnitude and distribution of ABFT in rejuvenated recycled asphalt mixtures at high magnification using Scanning Electron Microscopy (SEM) and Energy Dispersive X-ray Spectroscopy (EDS) and compare the results to those obtained using analytical models.

## 2. Research Objectives

The following are the main goals of the current research:

(1) To conventionally assess the ABFT in recycled asphalt mixtures, using analytical models;
(2) To measure ABFT in recycled asphalt mixtures, at the highest magnification, using SEM and EDS, and to check the validity of the ABFT assessed in step (1);
(3) To assess the consequence of recycled asphalt mixture and rejuvenator content on ABFT using analytical models, and image analysis utilizing SEM and EDS.

## 3. Materials and Methods

### 3.1. Aggregate

The virgin aggregate utilized in existing study was characterized in terms of conventional index properties, as presented in Table 3.

### 3.2. Asphalt Cement

The 60/70 pen-grade asphalt binder was acquired from the Attock refinery in Taxila, Pakistan, and its physical features are displayed in Table 4.

The aged asphalt binder, extracted from recycled asphalt mixture through the centrifuge method, was rejuvenated with 3%, 6%, 9%, and 12% waste engine oil (WEO) and characterized in terms of physical and rheological properties, as provided in Table 5 and Table 6.

The rheological properties of the virgin and rejuvenated recycled binder are presented in Table 6.

## 4. Research Methodology

**Figure 1:**
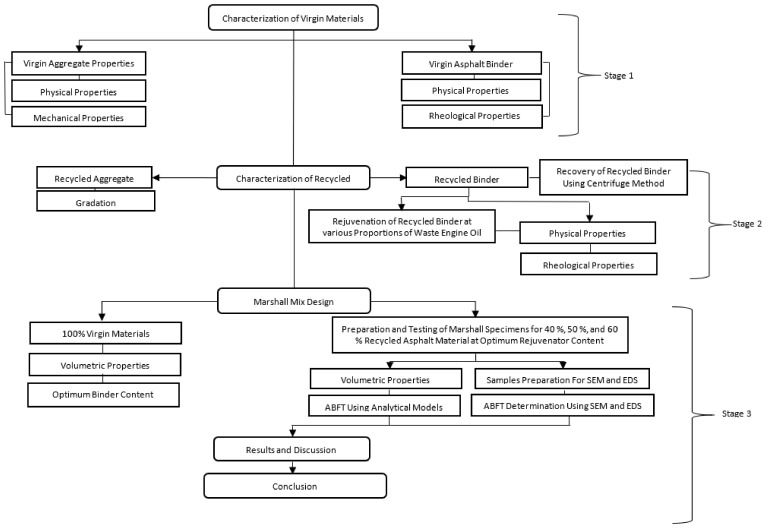
Research algorithm.

**Figure 2:**
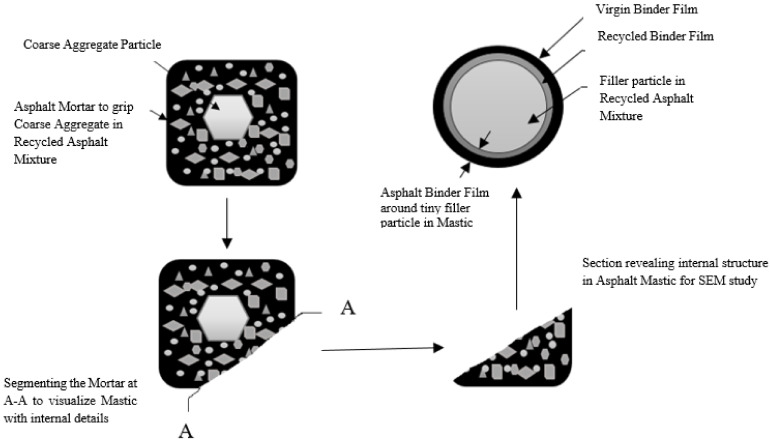
Specific research area for SEM study. A–A, Section to show internal details in mastic.

The research methodology is shown in Figure 1 and Figure 2, which comprises characterization of aggregate, virgin binder, and aged binder extracted from grinded recycled asphalt mixture, in terms of physical and rheological properties, respectively. Execution of the Marshall Mix Design for the conventional mix was implemented at 160 °C in order to conclude the optimum binder content (OBC) for virgin HMA. Marshall Mix Design was implemented for the composite mixtures containing 40% Recycled Mix + 60% Virgin Mix, 50% Recycled Mix + 50% Virgin Mix, and 60% Recycled Mix + 40% Virgin Mix, modified with 3%, 6%, 9%, and 12% waste engine oil (WEO) as rejuvenator (RJ). The optimum rejuvenator content (ORC) was determined on the basis of Marshall Stability and flow analysis, for each combination of recycled asphalt mixture, using OBC already obtained for the conventional mix. Additionally, ABFT was determined through conventional models, delivered in Table 2, and the conventionally predicted ABFT was actuated using SEM, and EDS.

## 5. Asphalt Mixture Design

**Figure 3:**
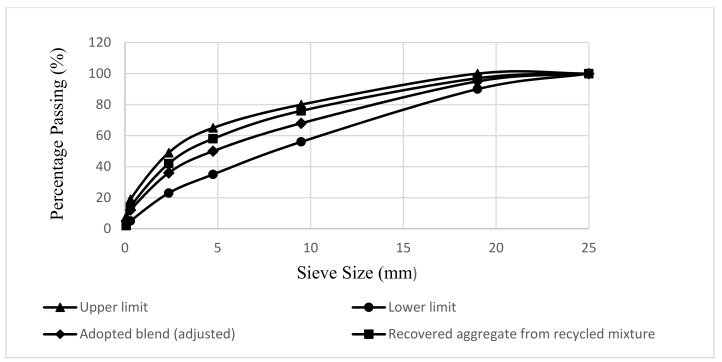
Grain size distribution curve of the adopted and recovered aggregate blend.

The virgin aggregate blend was produced following to the Asphalt Institute gradation (1994) for the Class-A asphalt wearing course, with the grain size distribution curve shown in Figure 3. The ring road, which was constructed in April 2010, acts as a bypass for heavy transport vehicles and facilitates traffic flow into Afghanistan. Due to severe rutting and fatigue cracking, the wearing course was dismantled in 2020 so that it could be rebuilt. As illustrated in Figure 3, the recycled asphalt materials were collected, and the aggregate mix was arranged with the same gradation as virgin aggregate. Figure 3 also shows the actual gradation of recycled aggregate recovered by an extraction test, where the gradation curve is on the finer side due to continuous aggregate collision in the centrifuge during extraction test and milling operation onsite. As a result, the collective aggregate blend, comprising both conventional and recycled asphalt mixture, was prepared, as shown in Figure 3, validating the Asphalt Institute (AI) gradation (1994).

Marshall Specimens for the conventional mix were prepared at varying binder concentrations and 160 °C mixing temperature, where the OBC was determined according to protocol, put forward in ASTM D1559. The volumetric properties for conventional mix design were obtained and offered in Table 7. An OBC of 4.12%, by weight of Marshall specimen, was concluded for the conventional mix at a mixing temperature of 160 °C. The mixtures met the lowest stability standard of 8 KN, with specified flow at the design circumstances. The bulk and theoretical specific gravities were 2.343 to 2.354 and 2.455 to 2.532, respectively. The 14% (minimum), 65% to 75%, and 3% to 5% standards were all fulfilled by the VMAs, VFAs, and VTMs, respectively.

The recovery of the recycled binder using the centrifuge method was done as per the procedure described in ASTM D 2172-95 [43]. During the centrifuge procedure for the recovery of recycled binder, a recycled asphalt mixture of 1200 g, equal to the weight of the Marshall specimen, was employed, yielding a recycled binder of 3.2% by weight. Mix design, confirming AI gradation, as shown in Figure 3, was carried out for recycled mixtures, containing 40%, 50%, and 60% recycled asphalt materials, rejuvenated at 3%, 6%, 9%, and 12% waste engine oil, respectively. The virgin aggregates were heated for 3 h at 180 °C. The fractions of the recycled asphalt mixture were spread in metal pans, according to the AASHTO R30 [44] standard, and then heated for 1 h at 160 °C. The European standard (EN 12697-35) [45] allows for 3 h of preheating. However, to avoid overheating and further ageing of the recycled binder, the preheating time was reduced to 1 h. After mobilizing recycled binder, the specified dosage of rejuvenator by weight of recycled binder was sprayed on the recycled asphalt mixture and diffused in recycled binder for 30 min. The virgin aggregate and binder were mixed separately at 160 °C. The virgin and rejuvenated recycled asphalt mixtures were mixed together at a mixing temperature of 160 °C, which is based on the temperature-viscosity relationship of the virgin and rejuvenated recycled binder. The following equation was used to compute the percentage of virgin binder required for mix design of recycled asphalt mixtures.

P_nb_ = {(100^2^ − rP_sb_)Pb/100(100 − P_sb_)}-{(100 − r)P_sb_/(100 − P_sb_)}—(Asphalt Institute, 1986)

where:

P_nb_ = Percent of new asphalt binder in recycled mix expressed as a whole number,

r = New aggregate expressed as a percent of the total aggregate in the recycled mix expressed as a whole number,

P_b_ = Percent estimated asphalt content of recycled mix assumed to be the same as that of 100% virgin HMA mix,

P_sb_ = Percent asphalt content of RAP.

In total, 72 Marshall Specimens containing 40%, 50%, and 60% recycled asphalt mixtures were prepared at already determined OBC as per ASTM D1559 [46]. Each aforesaid asphalt mixture was rejuvenated with 3%, 6%, 9%, and 12% waste engine oil, respectively, and evaluated according to ASTM D1559 [46]. Findings of the mix design were recorded in Table 8. A rejuvenator content of 3%, 6%, and 9% was concluded as optimum in terms of Marshall Stability and flow, for 40%, 50%, and 60% recycled asphalt mixtures, respectively.

The findings of the mix design for the recycled asphalt mixtures were used to quantify ABFT using analytical models specified in Table 2. The conventionally computed values of ABFT, based on analytical models, were verified by image analysis using SEM and EDS.

## 6. Quantifying Surface Area of Aggregates

The conventional estimation of ABFT was established on the basis of aggregates surface area, used in asphalt mix design. The percentage passing through each sieve was multiplied with specified surface area factors of aggregates, thus providing the total surface area for a specific sieve. The entire surface area (SA) in (m^2^/Kg) for the aggregate blend used in asphalt mix design was derived by adding all the areas calculated for each sieve. The aggregate blend’s total surface area was determined and shown in Table 9.

## 7. Image Analysis Using SEM and EDS

**Figure 4:**
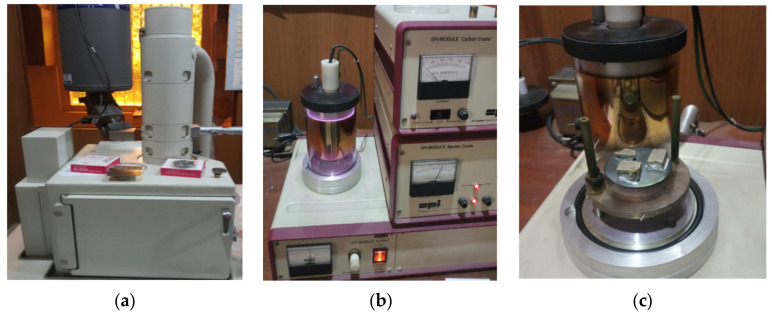
Components of SEM: (**a**) IT100 SEM, (**b**) gold coater, (**c**) specimen coating.

**Figure 5:**
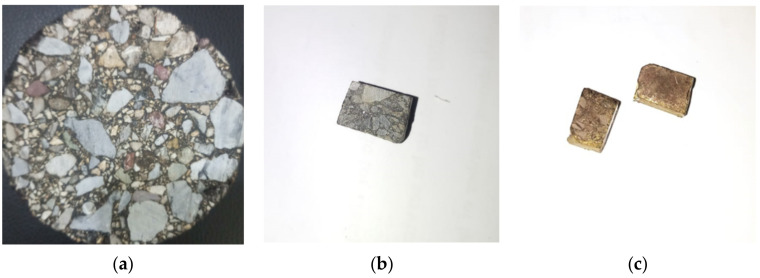
Sampling for microscopy: (**a**) Marshall sample, (**b**) uncoated specimen, (**c**) gold coated specimens.

**Figure 6:**
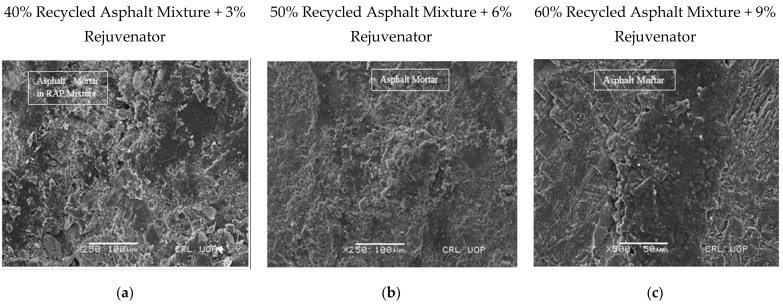
Microscopy of recycled mixtures: (**a**,**b**) Mortar at magnifying power ×250 and scale bar at 100 μm; (**c**) mortar at magnifying power ×500 and scale bar at 50 μm.

**Figure 7:**
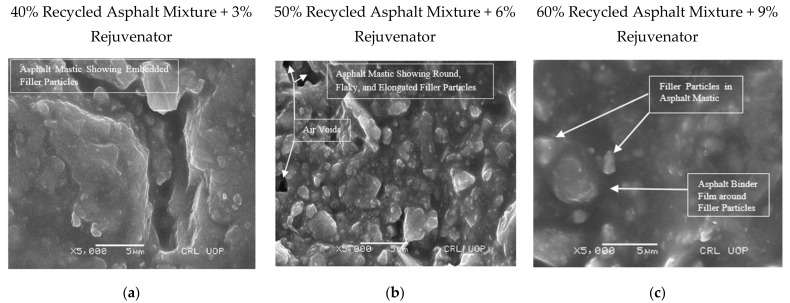
Microscopy of recycled mixtures: (**a**–**c**) Asphalt mastic at magnifying power ×5000 and scale bar at 5 μm.

**Figure 8:**
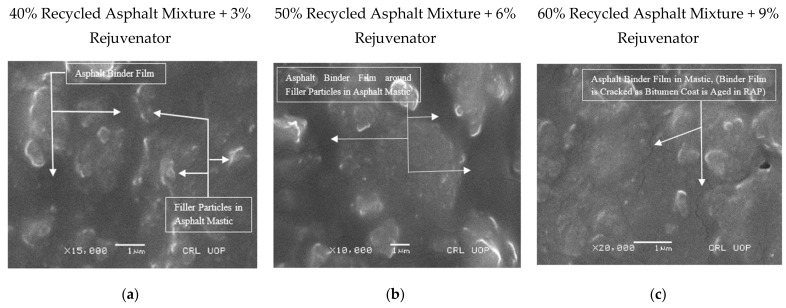
Microscopy of recycled mixtures: (**a**–**c**) Asphalt binder film in asphalt mastic at magnifying power ×10,000, ×15,000, and ×20,000, and scale bar at 1 μm.

**Figure 9:**
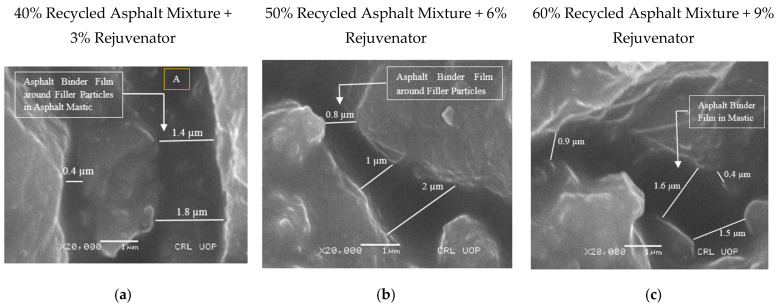
Microscopy of recycled mixtures: (**a**–**c**) Asphalt binder film in asphalt mastic at magnifying power ×20,000 and scale bar at 1 μm. A, Point for the elemental composition of binder film.

**Figure 10:**
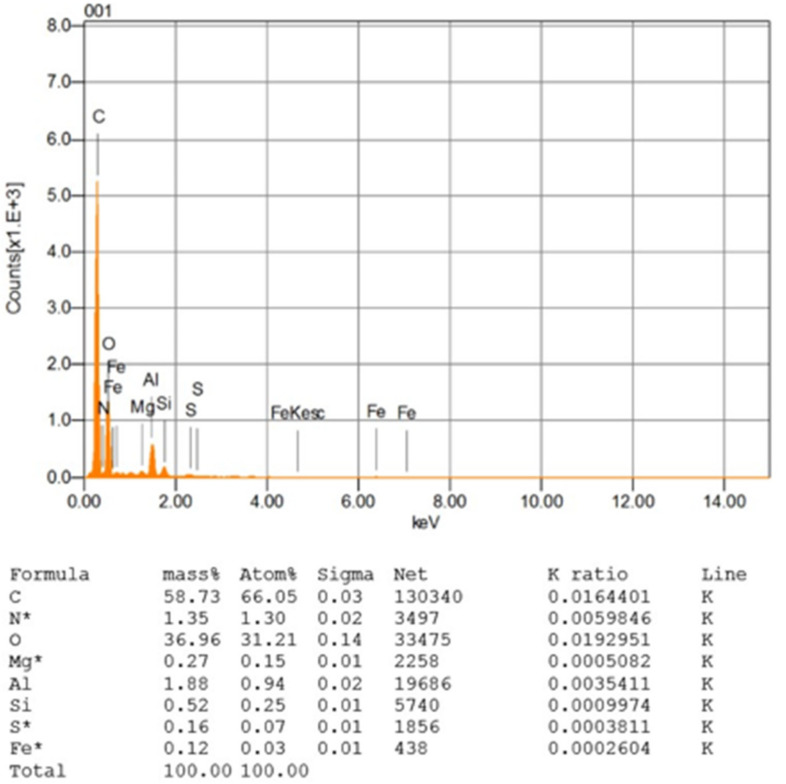
Elemental composition of asphalt binder film at point “A” in Figure 9a.

**Figure 11:**
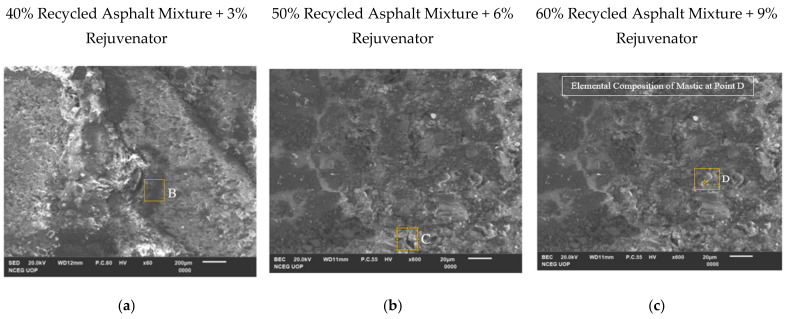

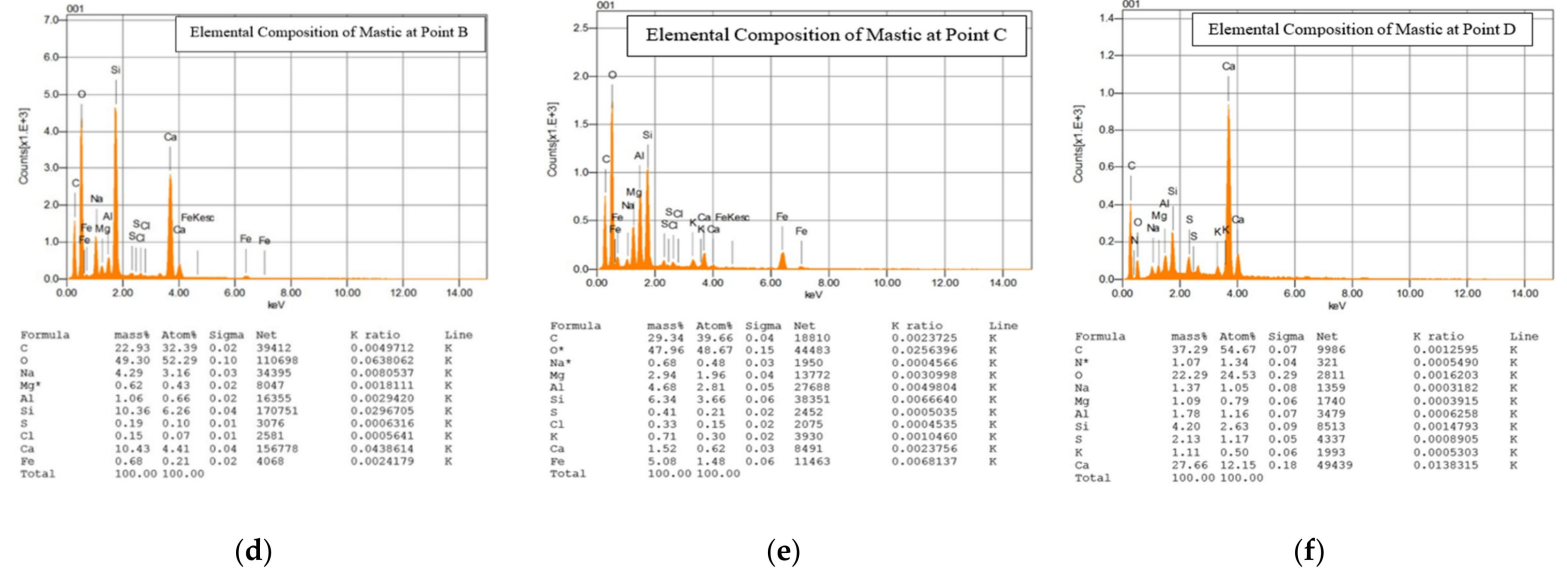
(**a**–**c**) SEM images of asphalt mastic in recycled asphalt mixtures. (**d**–**f**) Elemental composition of asphalt mastic at point “B”, “C”, and “D”. B, C, D: Points selected for elemental composition of asphalt mastic in recycled asphalt mixtures.

The current investigation used a JEOL JSM IT 100 scanning electron microscope (SEM) (JEOL Ltd., Tokyo, Japan) at a magnification range of ×250 to ×20,000, as illustrated in Figure 4a. The Marshall samples were sliced with a diamond saw to reveal the interior microstructure, and asphalt specimens of the appropriate sizes 7 mm × 7 mm × 5 mm and 10 mm × 6 mm × 6 mm were obtained, as revealed in Figure 5b,c. Figure 4b,c shows how a spot of interest was designated in the required specimens and coated with a thin film of gold (4 nm thick) using a sputter gold coater to make the exterior surface of the specified section conductive. As illustrated in Figure 5c, the metal-coated layer on the specimen surface was so thin that all of the micro details on the surface of the asphalt specimen were conserved and visible throughout the microscopic procedure. Figure 6, Figure 7, Figure 8, Figure 9, Figure 10 and Figure 11 show SEM images of asphalt mixtures containing 40%, 50%, and 60% recycled asphalt mixtures that have been rejuvenated with an optimum rejuvenator concentration of 3%, 6%, and 9%, respectively.

## 8. Results and Discussion

### 8.1. Analysis of ABFT Achieved through Analytical Models

In total, 72 asphalt samples, containing 40%, 50%, and 60% recycled asphalt mixtures, rejuvenated with an optimum rejuvenator content of 3%, 6%, 9%, and 12%, respectively, were prepared at already determined optimum binder content of 4.12%, by weight of Marshall specimen, and evaluated according to protocol presented in ASTM D1559. The findings were additionally utilized to assess ABFT for the aforesaid asphalt samples, by means of analytical models, and the outcomes of film thickness were provided in Table 10.

Under varied conditions of recycled asphalt mixture and rejuvenator content, the models delivered by Al-khateeb (FT_b_), SuperPave Series No. 2 (F_be_), Debao et al. (DA), and Road Note 19 (F) indicated an increasing drift in binder film thickness, ranging from 6.4 m to 13.5 m, as shown in Table 10. The variation in film thickness, with respect to recycled asphalt mixture and rejuvenator content, is accredited to the rise in recycled binder because of the rise in percentage of the recycled asphalt mixture and effective use of rejuvenator to mobilize recycled binder in recycled asphalt mixtures. However, the models offered by the Read and Whiteoak (T_F_) and Zaniewski et al. (T_f_) provided constant values of film thickness of 9.8 and 11.6 µm, respectively, without any change, with respect to recycled asphalt mixture and rejuvenator content. This is because essential components in the models were the density of asphalt binder and aggregate surface area, rather than temperature and compaction related parameters. As a result, following a specific ABFT limit was extremely challenging, as the models did not approve a precise range of film thickness, indicating uncertainty in traditional ABFT estimation.

### 8.2. Analysis of ABFT Achieved through SEM and EDS

SEM images of asphalt samples containing 40%, 50%, and 60% recycled asphalt mixture, rejuvenated with optimum rejuvenator concentration of 3%, 6%, and 9%, respectively, were captured at magnification powers of ×250 to ×20,000 to detect and measure ABFT. Entities as small as 0.2 µm were detected in the images captured by SEM and EDS. Figure 6, Figure 7, Figure 8, Figure 9, Figure 10 and Figure 11 provide the following observations based on the images.

Figure 6a–c shows SEM images of the 40%, 50%, and 60% recycled asphalt mixtures at 3%, 6%, and 9% ORC, respectively, taken at magnifications ×250, and ×500 with a bar scale of 100 and 50 µm, where the asphalt mortar, made up of bitumen, fine aggregate, and mineral filler, was revealed. The asphalt mortar was utilized to grasp large aggregates in position in the recycled asphalt mixture, ensuring structural integrity in the mixture. No voids at the aforementioned magnification were observed in the asphalt mortar because of the mobility of particles to voids in mortar during production and compaction during placement, but voids were observed at the interface between mortar and coarser particles, as shown in Figure 6a,c. When exposed to traffic and the environment, voids at the interface may form a weak zone that is prone to failures. Such voids can be managed by maintaining the required temperature during mixing and compaction of the mix, allowing the particles in the mix to be mobilized towards voids in the recycled mix.

Figure 7a–c displays images of the asphalt specimen, captured at magnification of ×5000 and a scale bar of 5 µm, which revealed asphalt mastic, comprising a mix of bitumen and mineral filler. Figure 7b shows flaky, elongated, and spherical filler particles in asphalt mastic, as well as air voids of 0.3, 0.7, and 1.4 micron sizes, which agrees with the findings of Karim et al. [10] but contradicts the assertion made by Kandhal and Chakraborty [11], that voids do not occur in asphalt mastic. Figure 8c also reveals a deviating binder film, gripping the filler particles in mastic.

The binder film began to appear in the mastic at magnifications of ×10,000, ×15,000, and ×20,000, with a scale bar of 1 m, as shown in Figure 8a–c. As seen in Figure 8c, the binder film cracked due to the rigidity of the recycled binder, indicating partial or no blending of the recycled binder with virgin binder or rejuvenator. As a result, the SEM sample was replaced ahead of time so that the binder film thickness in the rejuvenated recycled binder could be assessed. The SEM sample with the image in Figure 9c was used instead of the SEM sample with the image in Figure 8c.

Figure 9a shows clearly visible and quantifiable ABFT at a magnification of ×20,000 and a bar scale of 1 µm. The film thickness in the asphalt mixture, containing 40% recycled asphalt mixture at 3% rejuvenator content, as assessed by image analysis, varied from 0.4 to 1.8 µm, according to the experimental investigation. On the other hand, the analytically predicted ABFT varied from 6.4 to 11.6 µm, with the film thickness having a fair association with recycled asphalt mixture and rejuvenator content, as shown in Table 10.

According to SEM research, the ABFT in the asphalt mixture containing 50% recycled asphalt mixture at 6% rejuvenator content varies between 0.8 and 2 µm, as shown in Figure 9b. On the other hand, the traditionally computed values of model-based ABFT in the aforementioned recycled mixture ranged from 8.3 to 11.6 µm, with a strong relationship to recycled asphalt mixture content, rejuvenator content, and model type, as shown in Table 10.

Figure 9c shows the asphalt binder film, which was exhibited and measured at a magnifying power of ×20,000 and 1 µm bar scale in asphalt mixture containing 60% recycled asphalt mixture at 9% rejuvenator content. According to SEM research, the ABFT in the recycled asphalt mixture fluctuates from 0.4 to 1.6 µm. However, utilizing analytical models, the ABFT at the aforesaid circumstances fluctuates from 9.8 to 13.5 µm, with a strong association to recycled asphalt mixture content, rejuvenator content, and model type, as shown in Table 10.

According to SEM and EDS studies, the binder film thickness in recycled asphalt mixtures varies from 0.4 to 2 µm, with no correlation to recycled asphalt mixture or rejuvenator concentration, as shown in Figure 9. The results of SEM-based ABFT coincide with those of Elseifi et al. [18] and Karim et al. [10], but they contradict the analytical results of ABFT given in Table 1 and Table 10. As indicated in Table 10, traditional ABFT values based on analytical models range from 6.4 to 13.5 µm, with film thickness being a function of recycled asphalt mixture’s content, rejuvenator content, and model type. The analytically assessed values of ABFT, as per the current study, accord with the analytically assessed findings of ABFT shown in Table 1, but they contradict the aforementioned SEM-based findings of ABFT. Figure 9a–c indicates that the asphalt binder film appears as a separate entity that only exists as a non-absorbed and non-uniform binder, surrounding the mineral fillers in the asphalt mastic, occupying an approximate region of 0.4 to 2 μm among the filler particles. Figure 9a–c demonstrates that the asphalt binder film deviates by nature, which contradicts the assumption of a uniform binder film, used to develop the analytical models provided in Table 2.

According to the analytical analysis, the imprecise limit of film thickness and its dependence on recycled asphalt mixture and rejuvenator content exhibited a lot of inconsistencies in the conventional estimation of ABFT when compared to the SEM study. In terms of SEM analysis, the values of film thickness can be reliable because SEM images clearly illustrate what happens in the sample and what the probable range of ABFT is, as shown in Figure 6, Figure 7, Figure 8 and Figure 9.

Point “A” on the asphalt binder film was specified in Figure 9a to be used for the elemental composition of asphalt binder film to verify the existence of pure asphalt film. Figure 10 depicts the asphalt film elemental pattern, which exclusively contains bitumen elements, suggesting the presence of pure asphalt binder film.

Figure 11d–f illustrates the elemental components of asphalt mastic at points “B”, “C”, and “D” in Figure 11a–c, confirming that asphalt mastic is a blend of bitumen and mineral filler.

**Figure 12:**
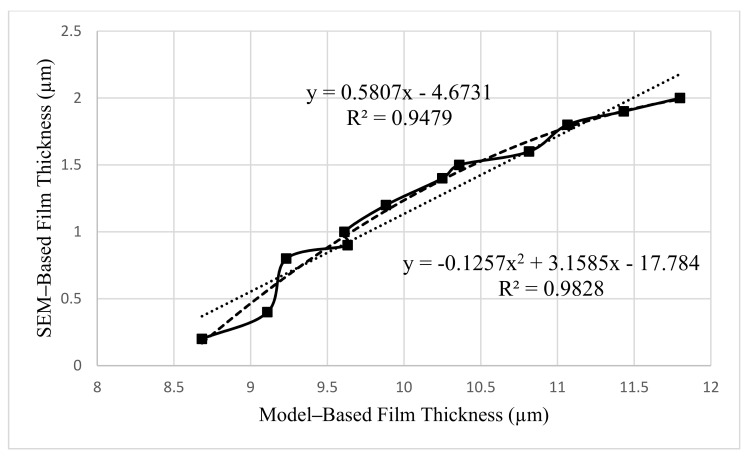
Relationship between SEM–based and model–based film thickness.

The association between SEM-based ABFT and average model-based ABFT, computed using analytical models in Table 10, is displayed in Figure 12 as a linear function with R^2^ of 0.9479 and a polynomial function of order 2 with R^2^ of 0.9828, indicating that the interaction is highly significant. The analytically analyzed outcomes of ABFT, as previously proven, are based on assumption-grounded models. These assumptions include sphere-shaped aggregates, undeviating ABFT on them, with no elucidation of the magnitude of compaction or porosity of the asphalt mixture, all of which could contribute to inaccuracies in traditional ABFT calculations. As a result of the expansion of ultra-modern expertise and SuperPave requirements, the critical need to study the concept of ABFT at the micro-level was encountered, revealing the association depicted in Figure 12, which will be useful in transforming traditionally assessed ABFT into SEM-based ABFT for the recycled asphalt mixtures.

## 9. Conclusions

The study’s main goal was to investigate the ABFT in recycled asphalt mixtures, utilizing micro-level procedures to verify the validity of the traditional method of estimating ABFT. The following are the conclusions attained:

- Using analytical models, the traditional method of estimating ABFT in recycled asphalt mixtures found that the film thickness is a function of the recycled asphalt mixture’s content, rejuvenator content, and model type. A substantial disparity in determining the ABFT was discovered for the same mix design. It is difficult to stick to a strict film thickness limit to be followed for adequate performance of asphalt paving mixtures.
- Using existing analytical models, the conventional estimation of ABFT in recycled asphalt mixtures, under all conditions of recycled asphalt mixture and rejuvenator content, revealed that the film thickness fluctuates from 6.4 to 13.7 µm, with a fair association to recycled asphalt mixture content and rejuvenator content to mobilize recycled binder. However, imaging analysis discovered that the ABFT for the above-mentioned circumstances fluctuates between 0.4 and 2 µm, with no correlation to recycled asphalt mixture and rejuvenator content.
- The asphalt binder film is a non-absorbed binder that occurs as a deviating stand-alone entity, exclusively surrounding the mineral fillers in the asphalt mastic, occupying an imprecise space of 0.4 to 2 µm among the filler particles.
- In the asphalt mixtures under consideration, air voids can be found at the interface between asphalt mortar and coarse particles, as well as within the asphalt mastic at the boundary of flaky and elongated filler particles, which may originate the weak zone.
- According to SEM analysis, the asphalt mortar is used to grip only coarse aggregate in the recycled asphalt mixtures. The asphalt mastic, on the other hand, was discovered to be an interlocking agent, binding fine aggregate in asphalt mortar.

## Figures and Tables

**Table 1:** Recommended analytically assessed values of ABFT.

ABFT (µm)	Reference	ABFT (µm)	Reference
9–10 µm @ 8 % air voids	Kandhal, and Chakraborty [11]	8 µm (average)	McLeod [12]
9–10 µm (min)	Kandhal et al. [13]	9–10 µm	Sengoz and Agar [14]
6–8 µm (min)	Kandhal et al. [13] Heitzman [15]	9–10 µm 7.5–9 µm	Sengoz and Agar [14] Oliver [16]
9–15.5 µm, (INDEX Model)			
8.5–13.5 µm, (VIRTUAL Model)	Heitzman [15]	9–11 µm	AlKofahi [17]

**Table 2:** Models to assess ABFT in HMA [10].

Analytical Models for Estimating ABFT in Microns (μm)	Reference	Analytical Models for Estimating ABFT in Microns (μm)	Reference
$T_{F}=\frac{b}{100-b}\times \frac{1}{\rho_{b}}\times \frac{1}{\mathrm{SA}}$	Read and Whiteoak [20]	$\mathrm{DA}=\frac{P_{\mathrm{be}}\times 1000}{\left(100-P_{b}\right)\times \gamma_{b}\times \mathrm{SA}}$	Debao et al. [21]
$T_{f}=\left[\frac{W_{b}}{\mathrm{SA}\times 1000}\right]\times G_{b}$	Zaniewski et al. [22]	$F=\frac{10^{6}P_{\mathrm{be}}}{\left(100-{\;P}_{b}\right)}\times \frac{1}{\mathrm{SA}}\times \frac{1}{\rho_{b}}$	Road Note 19TRL Ltd. [23]
${\;F}_{\mathrm{be}}=\frac{981\times P_{\mathrm{be}}}{\mathrm{SST}\times \left(100-P_{b}\right)}$	SuperPave Series No. 2 (SP-2) [24]	${\mathrm{FT}}_{b}=\frac{10^{5}P_{\mathrm{be}}}{P_{s}\times G_{b}\sum ({\mathrm{SAF}}_{i}\times P_{i})}$	Al-Khateeb [2]

**Table 3:** Characterization of the Margalla aggregate.

Property	Standard	Value	Property	Standard	Value
Los Angeles abrasion value, (%)	ASTM C131 [25]	23.8	Flakiness index, (%)	BS 933-3 [26]	5.53
Water absorption, (%)	ASTM C127 [27]	0.845	Elongation index, (%)	ASTM D4791 [28]	4.2
Fractured aggregates (Two faces), (%)	ASTM D5821 [29]	100	Soundness (Fine Aggregates), (%)	ASTM C88 [30]	3.74
Bulk density, (kg/m^3^)	ASTM C29 [31]	1547	Impact Value, (%)	BS 812 [32]	14.4
Voids (Uncompacted fine aggregates), (%)	ASTM C1252 [33]	47.6	Sand equivalent value, (%)	ASTM D2419 [34]	81.2
Petrography	ASTM C295 [35]	Innoc-uous	Alkali silica reactivity	ASTM C586 [36]	Innoc-uous

**Table 4:** Characterization of the Virgin asphalt binder.

Property	Value
Penetration (25 °C, 1/10th of mm), ASTM D5 [37]	63.6
Softening point, (°C), ASTM D36 [38]	48.5
Ductility, (cm), ASTM D113 [39]	102
Flash and fire point, (°C), ASTM C142 [40]	265 283

**Table 5:** Physical properties of the recycled binder.

Property	Recycled Binder	Recycled Binder at Various Rejuvenator Contents			
		3%	6%	9%	12%
Penetration at 25 °C, (1/10th of mm), ASTM D5 [37]	31	37	49	57	67
Softening point, (°C), ASTM D36 [38]	76	71	62	53	48
Ductility, (cm), ASTM D113 [39]	37	44	53	59	71
Flash point, (°C), ASTM C142 [40]	278	271	260	257	249
Fire point, (°C), ASTM C142 [40]	299	283	277	266	261

**Table 6:** Rheological properties of the virgin and recycled binder.

Property	Standard	Virgin Binder	Recycled Binder	Recycled Binder at Various Rejuvenator Contents			
				3%	6%	9%	12%
Viscosity (135 °C), (Pa.s)	ASTM D4402 [41]	0.626	0.743	0.710	0.662	0.557	0.513
Viscosity (165 °C), (Pa.s)	ASTM D4402 [41]	0.171	0.664	0.541	0.502	0.414	0.354
Complex Shear Modulus G*/Sinδ at 64 °C,10 rad/s, (kPa)	ASTM D6373 [42]	1.32	9.62				

**Table 7:** Design parameters for the conventional mix.

Specimen	Symbol	Unit	Mix Design Results				
Asphalt binder by total mix	P_b_	%	3.5	4	4.5	5	5.5
Bulk specific gravity of compressed mix	G_mb_	-	2.343	2.361	2.376	2.368	2.354
Theoretical specific gravity of loose mixt	G_mm_	-	2.455	2.463	2.478	2.491	2.532
Air voids in total mix	VTM	%	4.541	3.923	3.927	5.240	6.637
Voids in mineral aggregate	VMA	%	14.722	14.356	14.677	15.461	15.972
Voids filled with asphalt	VFA	%	69.539	73.672	72.822	66.373	58.163
Stability	S	KN	9.832	11.624	12.410	11.206	8.912
Flow	F	mm	3.5	2.8	2.4	3.3	4.5
Dust proportion ratio	DP	%	1.15	1.17	1.16	1.19	1.28

**Table 8:** Mix design outcomes of recycled asphalt mixtures at various rejuvenator contents.

Virgin Binder (%)	Recycled Binder (%)	Recycled Mix (%)	Virgin Aggregate (%)	Rejuven-Ator (WEO) (%)	G_mb_ (%)	G_mm_ (%)	VFA (%)	VMA (%)	A.V (%)	Stability (KN)	Flow (mm)
4.12	3.2	40	60	3%	2.440	2.53	69.64	11.72	3.55	14.816	2.30
				6%	2.391	2.51	64.85	13.49	4.74	14.104	2.902
				9%	2.384	2.55	53.16	13.74	6.43	12.322	3.514
				12%	2.405	2.54	59.06	12.98	5.31	11.157	4.236
4.12	3.2	50	50	3%	2.408	2.49	74.42	12.88	3.29	13.335	4.598
				6%	2.422	2.50	73.52	12.37	2.27	13.809	3.343
				9%	2.409	2.50	71.65	12.84	3.64	12.775	3.823
				12%	2.403	2.51	67.35	13.06	4.26	10.496	5.262
4.12	3.2	60	40	3%	2.403	2.52	64.44	13.06	4.64	12.249	6.445
				6%	2.411	2.51	69.10	12.77	3.94	12.913	6.033
				9%	2.422	2.49	77.92	12.37	2.73	14.212	3.739
				12%	2.403	2.53	61.55	13.06	5.01	13.237	5.987

**Table 9:** Total surface area of aggregate blend [10].

Sieve Size		Specification Limits (% Passing)	Target Blend (% Passing)	Surface Area Factor (m^2^/Kg)	Surface Area (m^2^/Kg)
Inch	mm				
1	25	100	100	0.41	0.410
3/4	19	90–100	95	-	0.410
3/8	9.5	56–80	68	-	0.410
#4	4.75	35–65	50	-	0.205
#8	2.36	23–49	36	0.82	0.295
#50	0.3	5–19	12	6.14	0.736
#200	0.075	2–8	5	32.77	1.638
Total Surface Area of Aggregate Blend, m^2^/Kg					4.104

**Table 10:** ABFT estimated using rejuvenated recycled asphalt mixtures.

Models Estimating Film Thickness (µm)	ABFT in Recycled Asphalt Mixtures at Rejuvenator Content of 3%, 6%, 9%, and 12%, (µm)											
	40% Recycled Asphalt Mixture				50% Recycled Asphalt Mixture				60% Recycled Asphalt Mixture			
	3%	6%	9%	12%	3%	6%	9%	12%	3%	6%	9%	12%
${\mathrm{FT}}_{b}=\frac{10^{5}P_{\mathrm{be}}}{P_{s}\times G_{b}\sum ({\mathrm{SAF}}_{i}\times P_{i})}$	8.9	10.4	10.8	11.2	9.3	9.7	10.2	10.5	10.3	10.6	11.2	11.8
${\;F}_{\mathrm{be}}=\frac{981\times P_{\mathrm{be}}}{\mathrm{SST}\times \left(100-P_{b}\right)}$	6.7	7.5	7.6	8.6	8.3	8.7	9.2	9.9	12.1	12.6	13.5	13.7
$\mathrm{DA}=\frac{P_{\mathrm{be}}\times 1000}{\left(100-P_{b}\right)\times \gamma_{b}\times \mathrm{SA}}$	6.4	7.2	7.7	8.3	8.0	8.3	8.8	9.3	9.8	10.3	10.7	11.5
$T_{F}=\frac{b}{100-b}\times \frac{1}{\rho_{b}}\times \frac{1}{\mathrm{SA}}$	9.8	9.8	9.8	9.8	9.8	9.8	9.8	9.8	9.8	9.8	9.8	9.8
$T_{f}=\left[\frac{W_{b}}{\mathrm{SA}\times 1000}\right]\times G_{b}$	11.6	11.6	11.6	11.6	11.6	11.6	11.6	11.6	11.6	11.6	11.6	11.6
$F=\frac{10^{6}P_{\mathrm{be}}}{\left(100-{\;P}_{b}\right)}\times \frac{1}{\mathrm{SA}}\times \frac{1}{\rho_{b}}$	8.7	9.5	7.6	8.3	10.4	11.2	11.9	12.7	11.3	11.5	11.8	12.4

## Data Availability

The complete data are available within the article.
